# Global biogeographic distribution of Bathyarchaeota in paddy soils

**DOI:** 10.1128/msystems.00143-23

**Published:** 2023-05-29

**Authors:** Shu-Dan Xue, Xing-Yun Yi, Hui-Ling Cui, Meng Li, Jing-Jing Peng, Yong-Guan Zhu, Gui-Lan Duan

**Affiliations:** 1 State Key Lab of Urban and Regional Ecology, Research Center for Eco-Environmental Sciences, Chinese Academy of Sciences, Beijing, China; 2 University of Chinese Academy of Sciences, Beijing, China; 3 Department of Plant and Environmental Sciences, University of Copenhagen, Frederiksberg, Denmark; 4 Archaeal Biology Center, Institute for Advanced Study, Shenzhen University, Shenzhen, Guangdong, China; 5 Shenzhen Key Laboratory of Marine Microbiome Engineering, Institute for Advanced Study, Shenzhen University, Shenzhen, Guangdong, China; 6 College of Resources and Environmental Sciences, China Agricultural University, Beijing, China; 7 Institute of Urban Environment, Chinese Academy of Sciences, Xiamen, China; E O Lawrence Berkeley National Laboratory, Berkeley, California, USA

**Keywords:** Bathyarchaeota, archaea, paddy soil, meta-analysis, co-occurrence network

## Abstract

Bathyarchaeota, known as key participants of global elements cycling, is highly abundant and diverse in the sedimentary environments. Bathyarchaeota has been the research spotlight on sedimentary microbiology; however, its distribution in arable soils is far from understanding. Paddy soil is a habitat similar to freshwater sediments, while the distribution and composition of Bathyarchaeota in paddy soils have largely been overlooked. In this study, we collected 342 *in situ* paddy soil sequencing data worldwide to illuminate the distribution patterns of Bathyarchaeota and explore their potential ecological functions in paddy soils. The results showed that Bathyarchaeota is the dominant archaeal lineage, and Bathy-6 is the most predominant subgroup in paddy soils. Based on random forest analysis and construction of a multivariate regression tree, the mean annual precipitation and mean annual temperature are identified as the factors significantly influencing the abundance and composition of Bathyarchaeota in paddy soils. Bathy-6 was abundant in temperate environments, while other subgroups were more abundant in sites with higher rainfall. There are highly frequent associations between Bathyarchaeota and methanogens and ammonia-oxidizing archaea. The interactions between Bathyarchaeota and microorganisms involved in carbon and nitrogen metabolism imply a potential syntrophy between these microorganisms, suggesting that members of Bathyarchaeota could be important participants of geochemical cycle in paddy soils. These results shed light on the ecological lifestyle of Bathyarchaeota in paddy soils, and provide some baseline for further understanding Bathyarchaeota in arable soils.

**IMPORTANCE:**

Bathyarchaeota, the dominant archaeal lineage in sedimentary environments, has been the spotlight of microbial research due to its vital role in carbon cycling. Although Bathyarchaeota has been also detected in paddy soils worldwide, its distribution in this environment has not yet been investigated. In this study, we conducted a global scale meta-analysis and found that Bathyarchaeota is also the dominant archaeal lineage in paddy soils with significant regional abundance differences. Bathy-6 is the most predominant subgroup in paddy soils, which differs from sediments. Furthermore, Bathyarchaeota are highly associated with methanogens and ammonia-oxidizing archaea, suggesting that they may be involved in the carbon and nitrogen cycle in paddy soil. These interactions provide insight into the ecological functions of Bathyarchaeota in paddy soils, which will be the foundation of future studies regarding the geochemical cycle in arable soils and global climate change.

## INTRODUCTION

Bathyarchaeota, formerly known as Miscellaneous Crenarchaeotal Group (MCG), were firstly discovered in hot spring (1) and have been characterized by high intragroup diversity containing 25 subgroups (2). Based on phylogenetic analyses, MCG was considered a novel archaeal phylum and proposed to name “Bathyarchaeota” (3, 4). Alternatively, the Genome Taxonomy Database (GTDB) has reclassified the phylum Bathyarchaeota to a class-level clade and renamed as Bathyarchaeia within the phylum of Thermoproteota. In addition to hot springs, Bathyarchaeota is widespread and abundant in various anoxic environments, such as sediments (5, 6), acid-sulfate springs (7), termite guts (8), and bioreactors (9).

Due to its highly abundant in various environments, Bathyarchaeota has been widely recognized as an important player in global geochemical cycling, especially in carbon cycling, which is closely linked to global climate change (2). Bathyarchaeotal multiple metabolic pathways have been inferred depending on genome information and isotope culture experiments. Evans et al. (10) recovered two bathyarchaeotal genomes belonging to Bathy-3 and Bathy-8, which contained genes encoding the methyl-coenzyme *M* reductase (MCR) complex, implying that Bathyarchaeota might be involved in methane metabolism. Bathyarchaeota are supposed to be capable to utilize various complex organic substrates, such as detrital proteins, aromatic compounds, carbohydrates, etc (11, 12). Through single-cell sequencing, genes encoding extracellular protein degrading enzymes were found in the incomplete bathyarchaeotal genomes obtained from marine subsea floor, indicating potential protein degrading capability (13). He et al. (3) recovered six Bathyarchaeota bins from a marine sediment sample collected from the Guaymas Basin in the Gulf of California, and the key genes involved in the reductive acetyl-CoA (Wood–Ljungdahl, WL) pathway were found in five bins, indicating that Bathyarchaeota had the potential of inorganic carbon fixation and acetate generation. In addition, genomic analysis demonstrated that Bathyarchaeota could degrade proteins, cellulose, chitin, and aromatic compounds for their own growth (3). Isotope culture experiments further demonstrated the important roles of Bathyarchaeota in sediment carbon cycle (14 - 16). Yin et al. (15) confirmed that Bathy-15 were active catabolic archaeal protein degraders in marine sediments. Moreover, Bathy-8 could utilize lignin as an energy source and bicarbonate as a carbon source, suggesting an organoautotrophic lifestyle of Bathyarchaeota (16). Our previous study found that adding fulvic acid to paddy soil significantly enriched Bathyarchaeota, which indicated that Bathyarchaeota could grow on fulvic acid or its metabolites as carbon sources (17). All these studies suggested that Bathyarchaeota might have diverse carbon metabolic pathways, including heterotrophic and autotrophic lifestyles, and different subgroups might be active degraders of different organic matters. In addition to carbon metabolism, Bathyarchaeota might also be involved in dissimilatory nitrite reduction to ammonium (18) and dissimilatory sulfate reduction (19). These studies greatly expanded our knowledge of ecological roles of Bathyarchaeota in sediments.

Besides diverse metabolic pathways, different bathyarchaeotal subgroups have specific environment preferences. Fillol et al. (20) collected bathyarchaeotal sequences and constructed a multivariate regression tree, indicating that salinity was the best explanatory variable for dissimilarity of bathyarchaeotal community. Bathy-1 and Bathy-8 dominated in saline sediments, while Bathy-11 and Bathy-5b dominated in freshwater sediments. Pan et al. (21) found that pH is the most important factor affecting the bathyarchaeotal community structure in mangrove wetlands and inferred that Bathy-6 preferred slightly acidic, high TOC, and subsurface environments. While Fillol et al. (22) demonstrated that Bathy-6 present in both planktonic and benthic habitats with a wide range of reducing conditions. Bathy-18 was found predominantly within the reduced environments, while Bathy-10 is the opposite (23). All these studies demonstrate that the habitat conditions shape bathyarchaeotal communities.

However, all these research progress on Bathyarchaeota mainly focuses on sedimentary environments. Paddy soil is usually flooded and similar to freshwater sediments that inhabit abundant Bathyarchaeota, while little attention has been paid to this distinctive habitat of Bathyarchaeota. Therefore, we hypothesized that Bathyarchaeota might be widespread and be important players for the biogeochemical cycle in paddy soils. Previously, some studies have found a high relative abundance of Bathyarchaeota in Italian paddy soils (24, 25). In contrast, a continental-scale survey of paddy soil in eastern China reported a low relative abundance of Bathyarchaeota (26). So far, the distribution and community composition of Bathyarchaeota in paddy soils has yet to be revealed. In addition, compared with sediments, the microbial community could be strongly influenced by plant growth and anthropogenic activities in paddy soil; thus, the distribution patterns of Bathyarchaeota and its subgroup might differ from that in sediments. Therefore, in this study, we collected *in situ* paddy soil sequencing data around the world, and aimed to illustrate the distribution patterns and subgroups compositions of Bathyarchaeota in paddy soils; to determine the environmental factors shaping the bathyarchaeotal community structure in paddy soils; to infer the potential functions of Bathyarchaeota in paddy soils according to the associations between Bathyarchaeota and other microorganisms.

## MATERIALS AND METHODS

### Publications collection and data set construction

**Fig 1:**
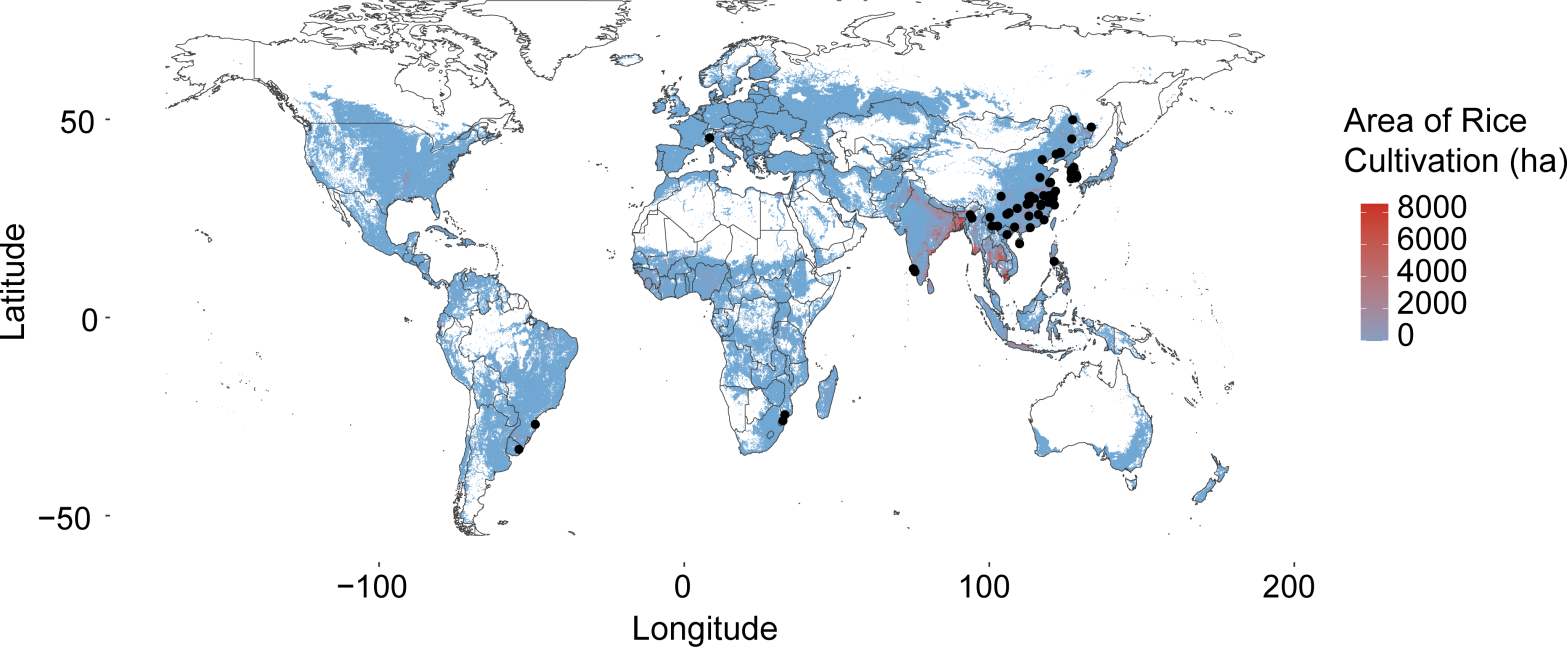
The location of sampling sites in this study. The black dots represented the sampling sites. The color of different areas on the map represented the area of rice cultivation. The closer the color to red meant the larger the area of rice cultivation. Data on area of rice cultivation came from https://www.mapspam.info/data/.

To investigate the distribution of Bathyarchaeota in paddy soil, we collected the publications about paddy soil microbial community by searching Google Scholar, PubMed, and Web of Science using “paddy soil” AND “microbial community” OR “microbial structure” as keywords (published before 1 September 2020). Then, we filtered the publications according to the following criteria: the soil samples should be collected from *in situ* soil or field trial, excluding microcosmic experiments and pot experiments; the amplicon primers should target archaea or both bacteria and archaea; raw data is available and can be downloaded from public databases according to the access number provided in the publications. After filtering, 23 publications (containing 342 paddy fields from nine countries) were included in this meta-analysis (Table 1). The sampling sites were mainly concentrated in Asia (China, South Korea, Philippines, Vietnam, and India), which cover the main rice production regions around the world (including Asia, Americas, Africa) (https://www.fao.org/faostat/en/#data/QCL/visualize) (Fig. 1, Table 1). Additionally, the samples used for the analysis included different water management phases. Details of these publication were shown in supporting information (Table S1). Climatic data for sampling sites was collected from WorldClim database (version 2.1, https://worldclim.org/) and sampling condition and soil physicochemical properties of each sample were collected from corresponding publication (Table S1).

**TABLE 1:** Sample sites and corresponding publications involved in this study

Sample_site	Number of samples	References
South Korea	43	(27 - 29)
Mozambique	16	(30)
Brazil	1	(31)
India	17	(32)
Vietnam	13	(33)
Italy	61	(24, 25)
China	170	(34 - 45)
Philippines	12	(46)
Uruguay	9	(47)

### Bioinformatics analysis

The raw data of each study was downloaded and independently processed with the same pipeline as the previous study (48) (Fig. S1). Briefly, nonbiological sequences were removed by Cutadapt (version 3.7). The trimmed sequences were denoised using DADA2 plugin in the Qiime2 (version 2022.2) (49). The feature table and amplicon sequence variant (ASV) sequences were obtained. Then the taxonomy classification of ASVs was performed using a pretrained Naive Bayes classifier and the q2-feature-classifier plugin based on the Silva 138 database. The unassigned ASVs and the ASVs belonging to mitochondria, chloroplasts, or eukaryotes were discarded. After data processing, samples with a sequence number of archaea of more than 500 were included in the meta-analysis. The relative abundance of archaeal lineages in these samples was tabulated as data set 1 (Table S2). The rarefaction of samples in data set 1 was performed in R (version 4.2.2) using “vegan” package. After rarefaction, the relative abundance of bathyarchaeotal subgroups in samples with Bathyarchaeota reads > 50 constituted the data set 2 (Table S3) and was included in the downstream analysis.

### Phylogenetic analysis

To investigate bathyarchaeotal community structure in paddy soil, the phylogenetic analysis was conducted to classify the Bathyarchaeota ASVs into different subgroups. The 2,030 ASVs belonging to Bathyarchaeota of data set 2 were dereplicated at 90% identity threshold and clustered into 80 operational taxonomic units (OTUs) with VSEARCH (version 2.7.0) (50). OTUs and reference sequences from Zhou et al. (2) were aligned by MAFFT (version 7.490) and trimmed by trimAl (version 1.4) (51). The remaining OTUs were classified into a reference tree (2) by RAxML (version 8.2.12) using “-f v -m GTRGAMMA” and the best tree was visualized by iTOL (https://itol.embl.de/).

### Statistical analysis

All statistical analyses and visualization were conducted in R (version 4.2.2) and Origin 2023. Species Abundance Distribution (SAD) was performed to investigate the abundance and pattern of archaeal lineages. The index of dispersion for each archaeal lineage was calculated as the ratio of the variance to the mean relative abundance multiplied by the occurrence to reflect the dispersion pattern of archaeal lineages (20). To investigate the effect of environmental factors on the microbial community, the samples were classified into different groups based on the mean annual precipitation (MAP) and the mean annual temperature (MAT). For MAP, the samples were divided into three groups (>2000 mm, >1000 mm and <2000 mm, <1000 mm). For MAT, the samples were divided into three groups (>20℃, >10℃ and <20℃, <10℃). Based on this classification, principal Coordinates Analysis (PCoA) based on Bray–Curtis distances and permutational multivariate analysis of variance (PERMANOVA) were carried out using “vegan” package. Random forest analysis was performed to rank the importance of factors influencing the abundance of Bathyarchaeota using “rfPermute” package with the option “ntree = 500, nrep = 1000”. And Wilcoxon rank sum test was used to further explore the preferred environment for Bathyarchaeota. A multivariate regression tree was constructed using “mvpart” and “MVPARTwrap” packages to identify the factors affecting the abundance of bathyarchaeotal subgroups with the option “xv = pick, xvmult = 100” (52).

### Co-occurrence network construction

Interactions between Bathyarchaeota and other microorganisms were inferred from the cooccurrence network. OTUs (at 90% identity threshold) with more than five sequences were used to calculate checkerboard score (C-score) and standardized effect size (SES) to analyze the randomness of the species distribution through “EcoSimR” package. Then, the Spearman correlations were calculated to construct cooccurrence network using “psych” package (53). The edges with spearman correlation > 0.6 and Benjamini–Hochberg adjusted *P* < 0.01 remained and used to construct the network by “igraph” package (54). The robust network was imported into Gephi (version 0.9) for visualization and property characterization.

## RESULTS

### The relative abundance of Bathyarchaeota in paddy soils

**Fig 2:**
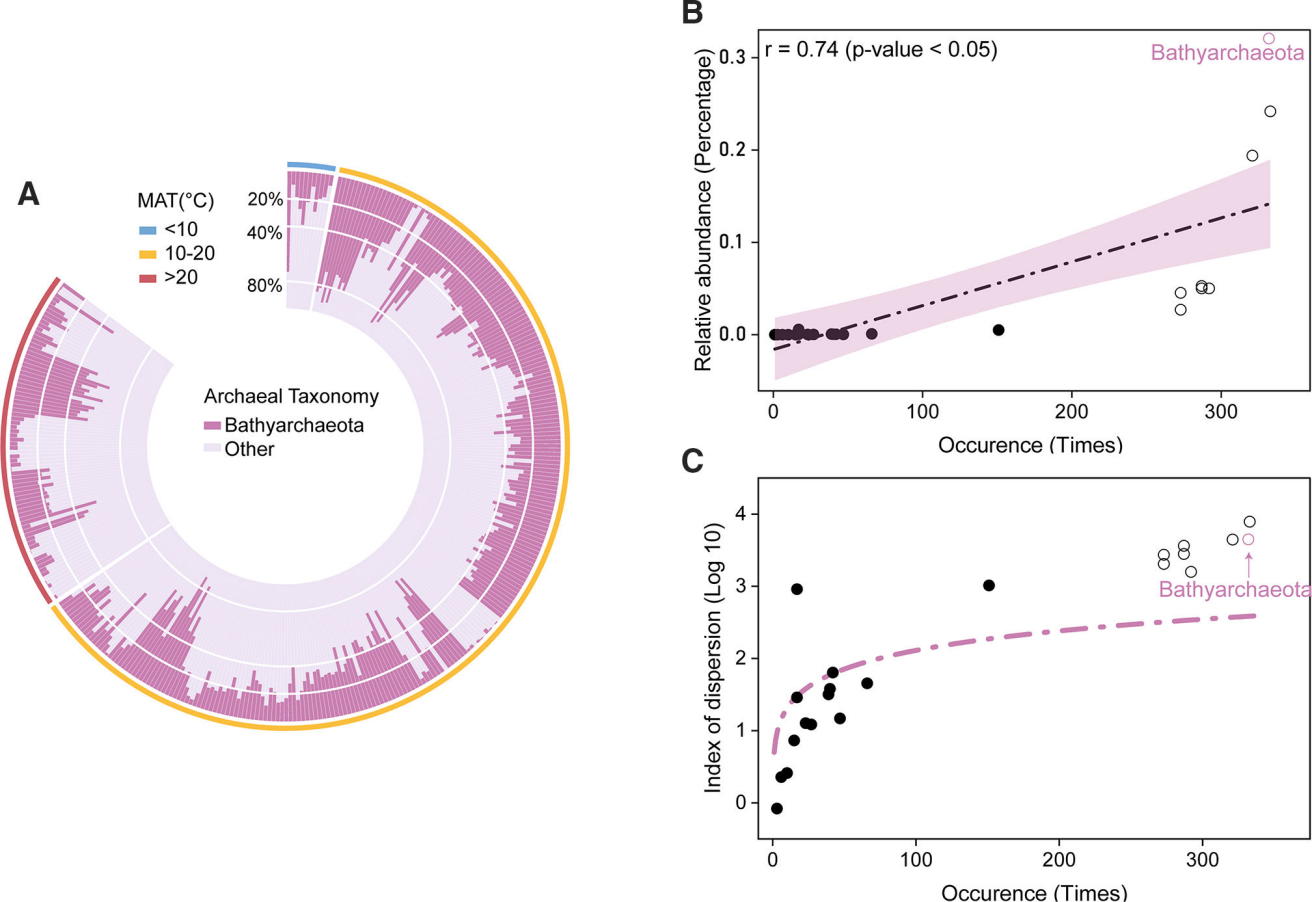
Distribution patterns of Bathyarchaeota in paddy soils. (**A**) Circular barplot showed the relative abundance of Bathyarchaeota within archaea in all samples. The samples were divided into three groups according to the annual mean temperature of the sample sites (> 20℃, > 10℃ and < 20℃, < 10℃. (**B**) The relationship between the relative abundance of all the archaeal lineages with their occurrence frequency. Hollow circles represent core lineages. (**C**) The index of dispersion against occurrence frequency. The line depicts the 2.5% confidence limit of the χ^2^ distribution. The point fell above of the line indicating a nonstochastic distribution.

In this meta-analysis, a total of 10,549 ASVs belonging to 24 archaeal lineages were obtained. Among these 24 archaeal lineages, Bathyarchaeota was the most frequently occurring lineage with the highest average relative abundance in paddy soils. Bathyarchaeota was detected in 332 samples, and its relative abundance ranged from 0.38% to 92.13% to the total relative abundance of archaea, with the average relative abundance of 32.07% (Fig. 2A and B). In addition, the relationship between the average relative abundance and occurrence frequency of archaeal lineages was analyzed to determine the ecological importance of Bathyarchaeota (Fig. 2). Results showed that there was a significant positive relationship between the average relative abundance against occurrence frequency (r = 0.74, *P* < 0.05). The index of dispersion against occurrence frequency plot were drawn to test whether these lineages followed a stochastic distribution (Fig. 2C). The point representing Bathyarchaeota fell above of the line depicted the 2.5% confidence limit of the χ^2^ distribution, indicating the distribution of Bathyarchaeota in paddy soils follows a nonstochastic process. In addition to Bathyarchaeota, *Nitrososphaeria* and *Methanosarcinia* were dominant archaea lineages in paddy soils with the relative abundances of 24.17% and 19.39% within archaea, respectively.

### The distribution patterns of Bathyarchaeota in paddy soils

**Fig 3:**
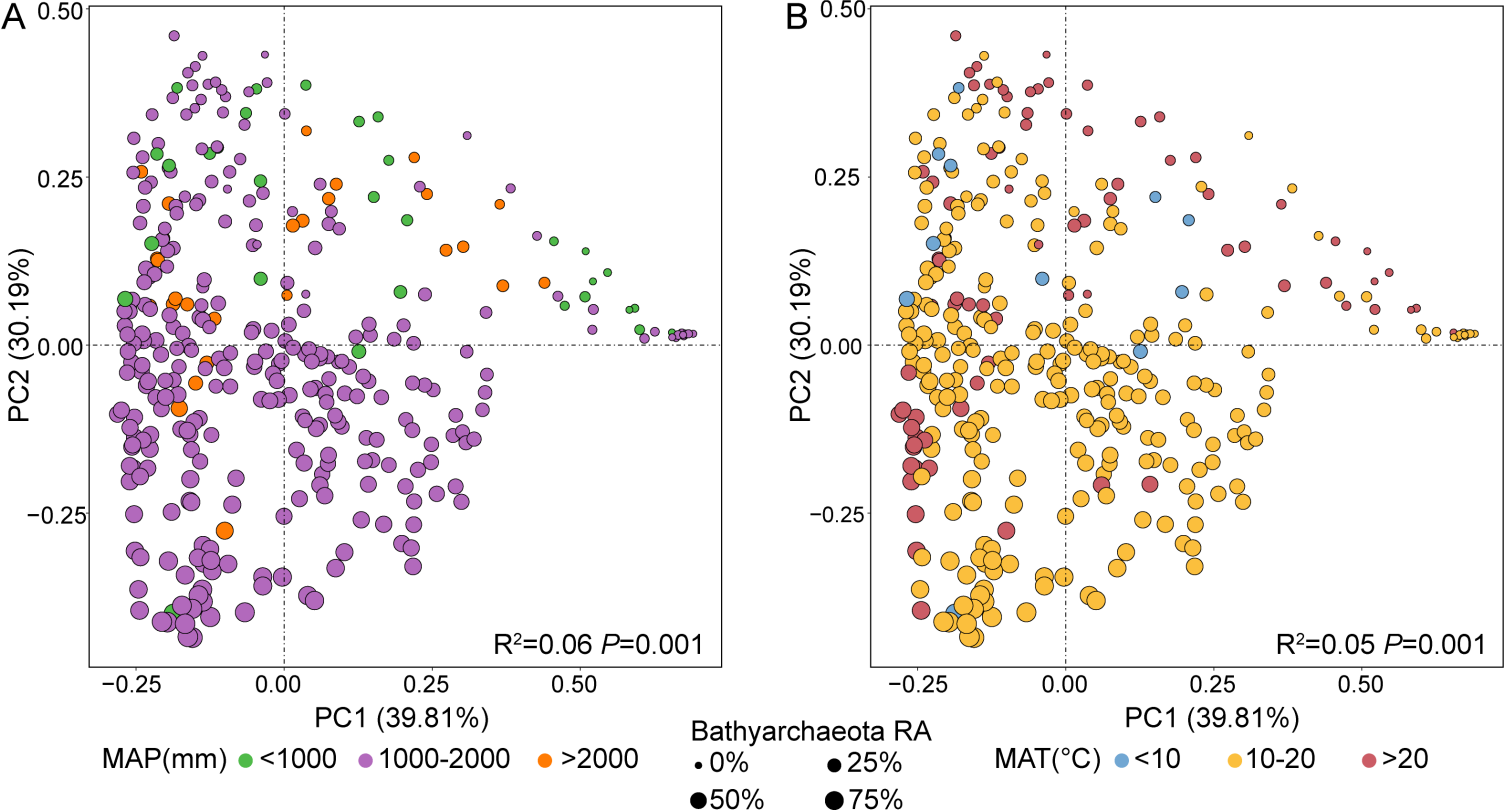
PCoA based on Bray–Curtis distances and PERMANOVA of archaeal community. Figure (**A**) and (**B**) colored the points according to the MAP and MAT, respectively. Samples were classified into different groups according to theMAP and the MAT. For MAP, samples were divided into three groups (>2000 mm, >1000 mm and <2000 mm, <1000 mm). For MAT, samples were divided into three groups (>20℃, >10℃ and < 20℃, <10℃).

To illustrate the influence of environmental factors on archaeal community structure, we classified all the samples into different categories according to climatic data and sampling site conditions (Table S1 and S2). Principal Coordinates Analysis based on Bray–Curtis distances was performed to investigate the difference in archaeal community structure among different samples (Fig. 3). PC1 and PC2 explained 39.81% and 30.19% of the total variance, respectively. As shown in Fig. 3, the archaeal community of all samples significantly separated in paddy soils with different annual precipitation, and also separated in paddy soils with different temperatures. In addition, the relative abundance of Bathyarchaeota also significantly separated according to annual precipitation and temperature. The samples with high relative abundance of Bathyarchaeota were concentrated in the regions with moderate temperature or medium rainfall (Fig. 3). The result of PERMANOVA indicated that the MAP explained the most variation (*R^2^* = 0.06, *P* = 0.001) and followed by the MAT (*R^2^* = 0.05, *P* = 0.001). Additionally, results illustrated that climatic factors more significantly influenced the archaeal community structure than agricultural activities, such as planting (*R^2^* = 0.03) or flooding (*R^2^* = 0.01).

**Fig 4:**
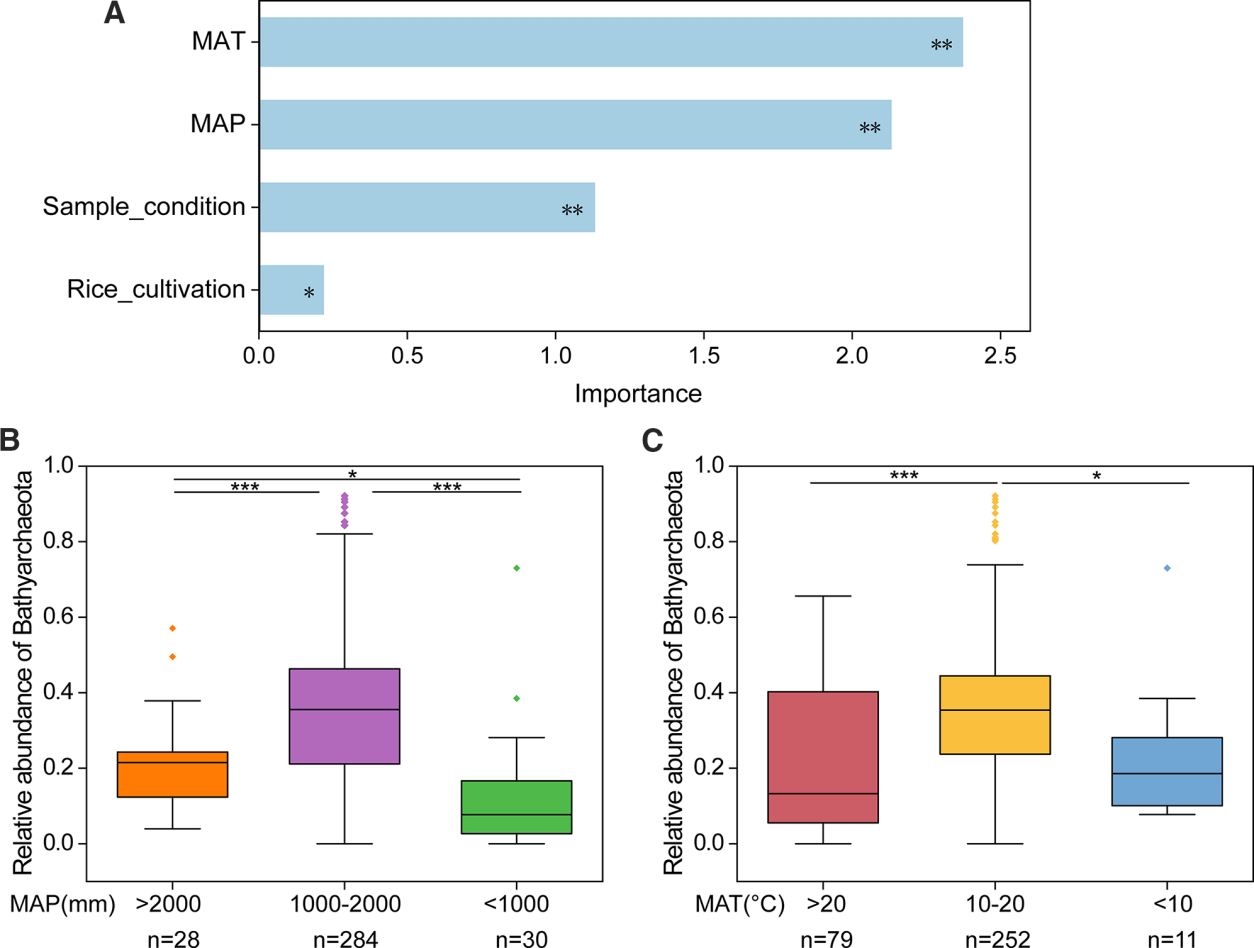
Environmental factors driving the abundance of Bathyarchaeota. (**A**) Random Forest Analysis revealed the importance of predictors on the relative abundance of Bathyarchaeota. (**B, C**) Wilcoxon rank sum test determined the significance of the difference in the relative abundance of Bathyarchaeota between different groups. The groupings of samples in Figure (**B**) and (**C**) were based on MAP and MAT, respectively.

To identify the key drivers for the abundance of Bathyarchaeota, random forest analysis was carried out to evaluate the importance of predictors on the relative abundance of Bathyarchaeota (Fig. 4A). MAT was found to be the most important predictor (IncNodePurity = 2.37) followed by MAP (IncNodePurity = 2.13). Compared with climatic factors, the agricultural activities, such as rice cultivation and sampling condition had less effects on the abundance of Bathyarchaeota (Fig. 4A). This result was consistent with the conclusion obtained from the previous PCoA analysis (Fig. 3). Wilcoxon rank sum test was performed to further characterize the environmental preference of Bathyarchaeota. Based on MAP classification, the relative abundance of Bathyarchaeota was significantly higher in samples with MAP between 1000 mm and 2000 mm (32.72%) than in those with MAP higher than 2000 mm (20.96%) and less than 1000 mm (12.30%) (*P* < 0.001) (Fig. 4B). For MAT, the relative abundance of Bathyarchaeota was highest under moderate temperature conditions (>10℃ and <20℃) (35.82%) than under high (>20℃) or low (< 10℃) temperature conditions (*P* < 0.01) (Fig. 4C, Fig. 2A). These results indicate that Bathyarchaeota prefer temperate environments.

### The biogeography of Bathyarchaeota subgroups in paddy soils

**Fig 5:**
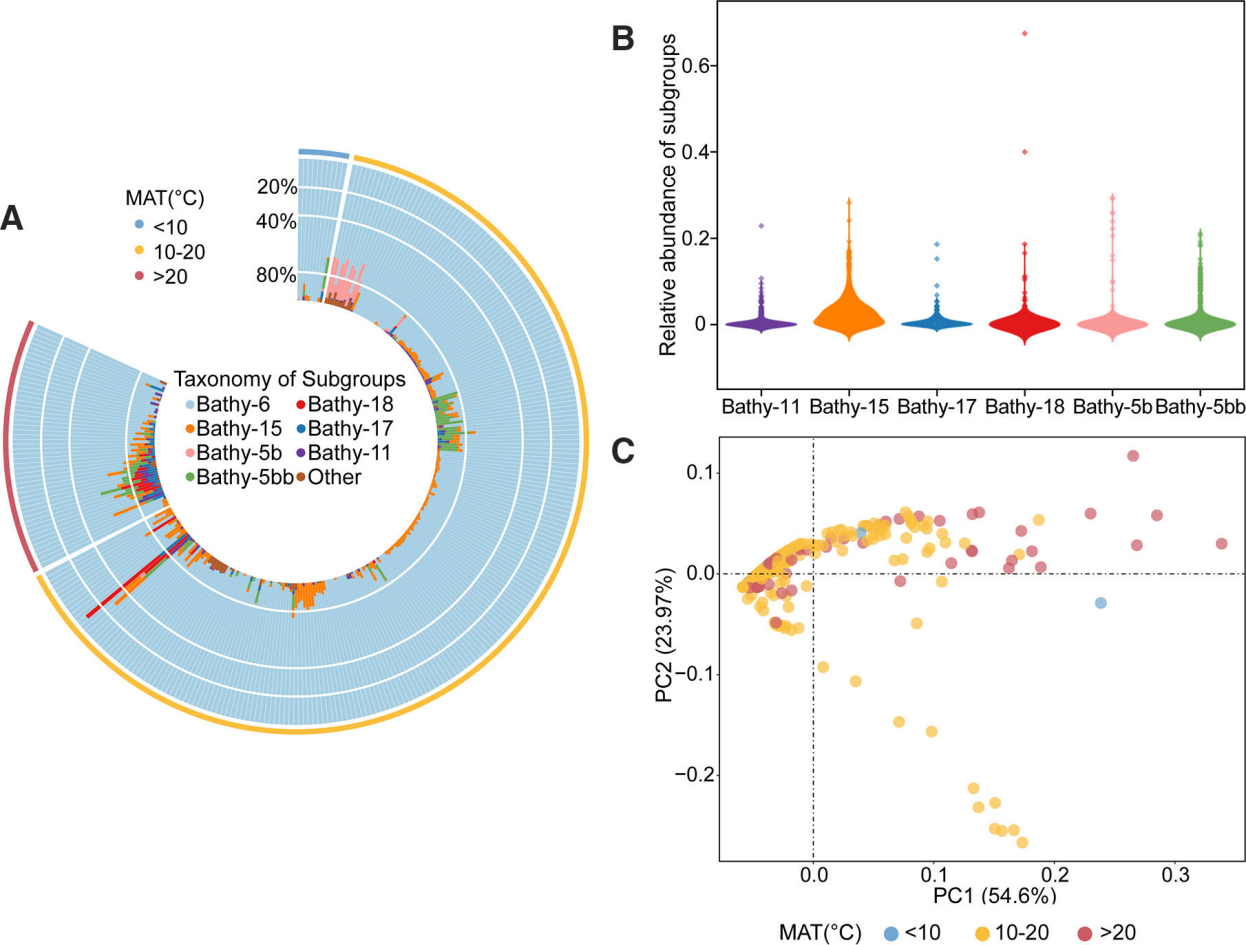
The composition pattern of bathyarchaeotal subgroups in paddy soils. (**A**) The relative abundance of bathyarchaeotal subgroups within Bathyarchaeota. (**B**) The abundance of the remaining subgroups except Bathy-6. (**C**) PCoA based on Bray–Curtis distances and PERMANOVA of bathyarchaeotal community. The points colored according to MAT.

There were 80 OTUs (clustered by 2,030 ASVs at the threshold of 97% similarity) belonging to Bathyarchaeota, belonging to 11 subgroups according to the reference tree constructed by Zhou et al. (2) (Fig. S2). Among these 80 OTUs, 40 OTUs were identified as Bathy-6, which was the overwhelmingly dominant subgroup in paddy soil, accounting for 91.67% of the whole bathyarchaeotal community with a relative abundance of 7.23% ~ 100% (Fig. 5A). In addition to Bathy-6, there were six other subgroups with relative abundance higher than 0.5%, accounting for 5.3% of bathyarchaeotal community (Fig. 5B). Bathy-15 was the second abundant subgroup which was detected in 192 samples with an average relative abundance of 3.01%.

PCoA analysis illustrated that all samples were mainly distributed along PC1 axis which explained 54.6% of the total variance (Fig. 5C). Both MAT (*R^2^* = 0.02, *P* = 0.012) and MAP (*R^2^* = 0.02, *P* = 0.004) significantly influenced the bathyarchaeotal community structure. We further conducted multivariate regression tree analysis to explore the effect of climatic factors and soil properties on distribution patterns of bathyarchaeotal subgroups. The result was presented as a four-leaf tree, explaining 43.7% of the total variance (Fig. S3A), and MAT was further identified as the key driver shaping bathyarchaeotal subgroups. Since MAT might covaries with the other environmental variables and a high proportion of Bathy-6 might weaken the abundance changes of the other subgroups, the specific drivers for different subgroups were further explored (Fig. S3B, Fig. S4). As shown in Fig. S3B, the relative abundance of Bathy-6 was significantly influenced by MAT (*R^2^* = 0.282), and the samples were clustered into two groups according to temperature 27.18℃. Bathy-6 was more abundant (with an average abundance of 93.35%) when MAT lower than 27.18℃ (*n* = 274), this result is confirmed by Wilcoxon sum test (*P* < 0.001). MAP was another important factor for the composition of bathyarchaeotal subgroups. Bathy-11, Bathy-17, Bathy-18, and Bathy-5bb were more abundant in paddy soils with higher MAP (Fig. S4). In addition to climatic factors, soil pH significantly influenced the abundance of Bathy-5b, Bathy-5bb, and Bathy-15.

### Interactions between Bathyarchaeota and other microorganisms

**Fig 6:**
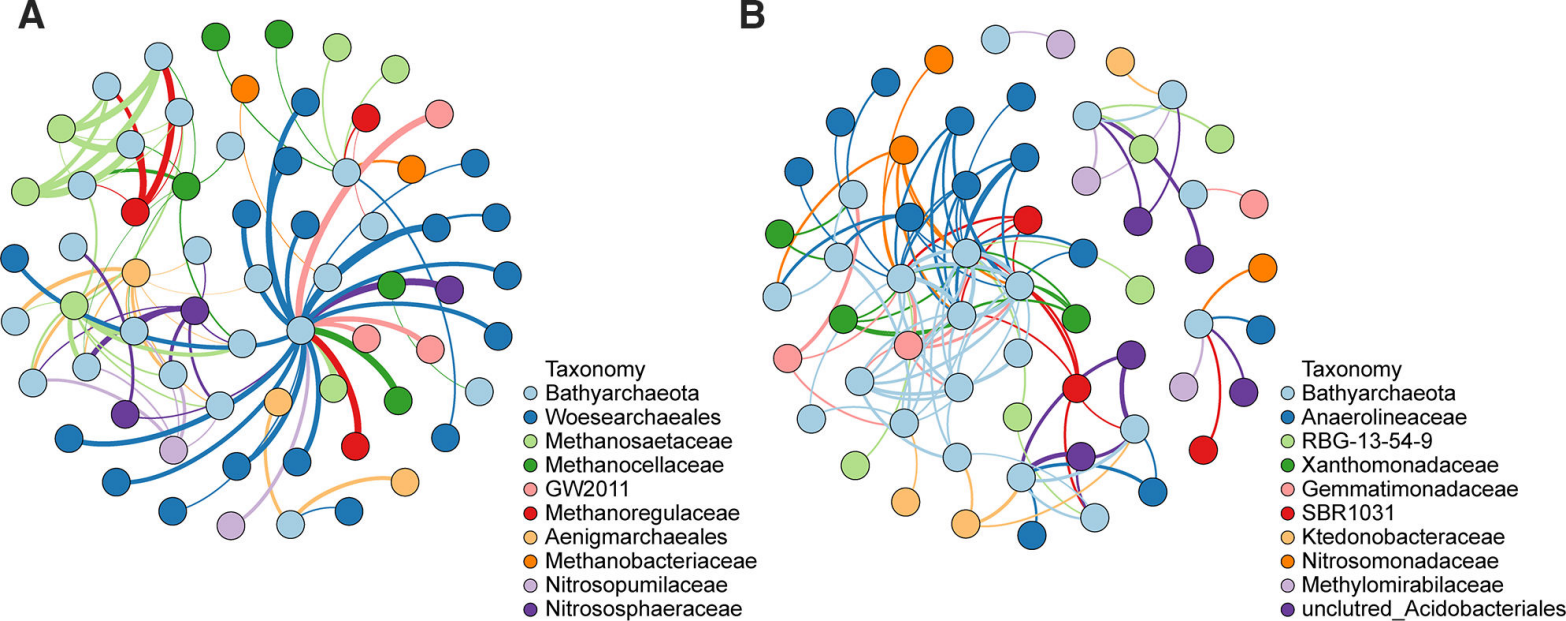
Co-occurrence network analysis based on Spearman correlations. (**A**) Co-occurrence network between Bathyarchaeota and other archaea. (**B**) Co-occurrence network between Bathyarchaeota and bacteria. Top nine microorganisms connected with Bathyarchaeota were shown in the network.

We constructed robust co-occurrence networks based on Spearman correlations (a coefficient > 0.6 and *P_FDR_* < 0.01) to preliminarily understand the potential interactions between Bathyarchaeota and other microorganisms. Firstly, the C-score was calculated to evaluate the co-occurrence patterns and stochasticity of archaeal community (54). We observed a C-score value of 278.68, which was significantly higher than simulated C-score (C-score_sim_ = 257.61, *P* < 0.001). The result rejected the null model hypothesis and confirmed that the archaeal community was a nonrandom and segregated co-occurrence pattern. The archaeal network was composed of 181 nodes and 1,052 undirected edges, which was classified into 18 modules with a module index of 0.56 (values > 0.4 suggest that the network has a modular structure) (Fig. S5A). Among all the archaeal lineages, Bathyarchaeota was the second most abundant node (16.02%), occurring in six major modules (Fig. S5B). To more clearly demonstrate the potential ecological niches of Bathyarchaeota, we filtered and retained the edges between Bathyarchaeota and the top 10 archaeal lineages (Fig. 6A). Within this network, Bathyarchaeota exhibited the closest connection with *Woesearchaeales*. In addition, methanogens, such as *Methanosaetaceae*, *Methanocellaceae*, *Methanoregulaceae,* and *Methanobacteriaceae*, had nonrandom associations with Bathyarchaeota. Moreover, Bathyarchaeota also co-occurred with ammonia-oxidizing archaea (*Nitrosopumilaceae* and *Nitrososphaeraceae*). After calculating the C-score, a nonrandom co-occurrence network between bathyarchaeotal and bacterial OTUs was also constructed (Fig. 6B). There were 377 edges connecting Bathyarchaeota and bacteria. Among them, *Anaerolineaceae* showed the most associations with Bathyarchaeota, followed by *RBG-13-54-9*.

## DISCUSSION

### Bathyarchaeota is widespread and abundant in paddy soils

Paddy soil is one of the major types of global land use (Fig. 1), and long-term flooding during rice cultivation makes it an important source of methane emissions (55). At the same time, paddy soil is an important carbon sink with long-term stable carbon sequestration (56). Hence, the carbon cycling in paddy soil has been a hot research topic. Bathyarchaeota, known as important players in the carbon cycle of environments with high carbon content, such as marine ecosystems and terrestrial permafrost (57), has been found to be abundant in paddy soil (24, 58). We thus inferred that members of Bathyarchaeota might be involved in paddy soil carbon metabolism. Therefore, we collected sequencing data from 342 *in situ* paddy soil samples worldwide to provide a primary understanding of the distribution and composition patterns of Bathyarchaeota at the global scale.

We analyzed the SAD (species abundance distribution) of the observed 24 archaeal lineages in paddy soil (Fig. 2B and C), which could be divided into two groups based on their frequency of occurrence and average abundance (20). One group was rare/less abundant satellite lineages, which were detected in less than 160 samples; another group was persistent/abundant core lineages consisting of 8 lineages which were detected in more than 270 samples. Bathyarchaeota were included in the persistent/abundant group, and which was the most frequently occurring lineages with the occurrence of 97% (observed in 332 samples of total 342 samples analyzed) (Fig. 2B). In addition, Bathyarchaeota shows high abundance in paddy soils, with an average relative abundance of 32.07%, which is similar to the abundance in the sediments (20). These results suggest that Bathyarchaeota is the core archaeal lineage in paddy soils. In this study, high abundance of Bathyarchaeota is observed in both flooded and unflooded paddy soils, with average relative abundance of 27.78% and 37.07% (Fig. 2), respectively. These results suggest that Bathyarchaeota was also dominant in aerobic paddy soil, and broadened previous prescription that Bathyarchaeota are generalists in anaerobic environments such as sediments (5), peatland (59), and anaerobic digestion system (9), which will expand our understanding of Bathyarchaeota.

The high diversity of Bathyarchaeota makes it interesting to understand the distribution patterns of different subgroups, which can contribute to revealing ecological functions of Bathyarchaeota. We identified 2,030 bathyarchaeotal ASVs in this study, and which were classified into 11 subgroups. Among these subgroups, Bathy-6 was the most predominant subgroup in all samples, this result is consistent with previously observed paddy soil archaeal community of the Yunhe terrace (58). The high abundance of Bathy-6 in paddy soils might result from the presence of genes encoding superoxide dismutase in genomes of Bathy-6 recovered from terrestrial environments (60). These findings suggest that the lifestyle of Bathyarchaeota might have changed in terrestrial and marine ecosystems and imply the metabolic pathway of Bathy-6 in paddy soils might be different from other members in marine sediments. In addition, Bathy-15 and Bathy-5bb were two other abundant subgroups in paddy soils with average relative abundance more than 1% (Fig. 5B), while their abundance was much lower compared to those detected in freshwater sediments (5, 59). However, Bathy-1 and Bathy-8, which are Bathyarchaeota indicator lineages of marine sediments (20), were almost undetected in paddy soils analyzed in this study. Based on these results, our study demonstrated that bathyarchaeotal community structure in paddy soils are distinctive to those in sediments.

### Climatic factors significantly influence bathyarchaeotal community

The relative abundance of Bathyarchaeota ranged from 0.38% to 92.13% within archaea, and this excessive variation among different samples made it is interesting to investigate the variation mechanisms and to identify the key factors regulating bathyarchaeotal abundance. Compared with sediments, the microbial community in paddy soil is more susceptible to agricultural activities, plant roots and climatic factors. Therefore, we collected the climatic data of sampling sites, soil properties and soil condition at the time of sampling from corresponding studies (Table S1) to perform random forest analysis and multivariate regression tree analysis. Results demonstrated that MAT and MAP strongly affected the archaeal community structure. These climatic factors have a long-term regulation of microbial activity and elements cycle, and have been generally considered to be important factors affecting microbial assembly (26, 61). Bathyarchaeota, as the core archaeal lineage in paddy soils, was also strongly influenced by MAT and MAP. We conjectured that the effect of MAT on the abundance of Bathyarchaeota was partly caused by variation in the abundance of Bathy-6, which is the most predominated subgroup in all the samples. Among all these factors, the proportion of Bathy-6 was only influenced by MAT (Fig. 3B). This result is in agreement with previous study (62), which found that high abundance was observed in samples with moderate temperature. Bathy-11, Bathy-17, Bathy-18, and Bathy-5bb were mainly influenced by MAP (Fig. S4). Surprisingly, the abundance of these subgroups, which are abundant in anoxic environments, was not affected by water management in paddy soils. In flooded paddy soil, rice aerenchyma can transport atmospheric oxygen to the rhizosphere, resulting in a microoxic environment. Thus either flooded or unflooded paddy soils are not anoxic environments; however, no genes involved in oxygen-dependent pathways have been detected in these subgroups so far. Thus, the abundance of these subgroups might depend on the gradient in reduction caused by abundant rainfall (34).

### Bathyarchaeota highly connects with microorganisms involved in carbon and nitrogen metabolism

Bathyarchaeota have been identified as complex organic matter degraders and participants in methane metabolism in sediments (10). However, the ecological roles and niches of Bathyarchaeota in paddy soil are still unclear. We performed co-occurrence network analysis to reveal the syntrophic interactions between Bathyarchaeota and other microorganisms, through which we can infer the ecological roles of Bathyarchaeota in paddy soils (Fig. 6).

Interestingly, highly frequent associations between Bathyarchaeota and *Woesearchaeales* were found in the co-occurrence network of archaeal lineages. Recent studies illustrated that *Woesearchaeales* are widespread in both terrestrial and marine ecosystems which are classified in the GTDB database as Woesearchaeota (63, 64). According to the analysis of metagenome-assembled genomes, *Woesearchaeales* exhibit conspicuous metabolic deficiencies, suggesting an anaerobic syntrophic lifestyle of *Woesearchaeales* (64, 65). This syntrophic lifestyle is consistent with our result, that *Woesearchaeales* have the frequent connections with other archaea (Fig. 5B). The co-occurrence of Bathyarchaeota and *Woesearchaeales* implied that *Woesearchaeales* might obtain intermediate products from the degradation of organic matter by Bathyarchaeota. For example, detrital proteins may be degraded to amino acids by extracellular peptidases encoded by Bathyarchaeota (13), which could be then used by *Woesearchaeales* for their own growth (64). In addition, Bathyarchaeota have close relationship with methanogens, such as *Methanosaetaceae* (known as *Methanotrichaceae*), *Methanocellaceae*, *Methanoregulaceae,* and *Methanobacteriaceae*. This result is consistent with the findings in the marine sediment (66) and terrestrial environments (62). Among these methanogens, *Methanosaetaceae* can directly use acetate as substrate, while hydrogen-consuming methanogenic archaea (*Methanocellaceae*, *Methanoregulaceae* and *Methanobacteriaceae*) prefer to utilize oxidation products of acetate (53, 67) to produce methane. Recent studies demonstrated that Bathyarchaeotal genomes encode a series of phosphate acetyltransferase (Pta) and acetate kinase (Ack) (3) or acetyl-CoA synthetase (Acd) for acetate production (18). Potential acetogeneic pathway of Bathyarchaeota might explain its close association with methanogens. Furthermore, the co-occurrence patterns can also result from overlapping ecological niches between microorganisms instead of syntrophy (68). Previous study revealed the presence of genes encoding the methyl-coenzyme *M* reductase (MCR) complex essential for methanogenesis (10), suggesting that Bathyarchaeota might produce methane as well as methanogens. Since the relationships between Bathyarchaeota and methanogens recurred in different modules of the network (Fig. S5), we preferred to consider a syntrophy between these two microorganisms. Besides archaeal network, we also constructed the network between Bathyarchaeota and bacteria (Fig. 6B). We found that *Anaerolineaceae* was the bacterial lineage that had the most frequent nonrandom associations with Bathyarchaeota (Fig. 6B). It has been reported that members of *Anaerolineaceae* are typical complex organic matter degraders under anoxic condition (69), implying that Bathyarchaeota might be involved in the carbon cycle in paddy soils.

In addition, the co-occurrence associations between Bathyarchaeota and ammonia-oxidizing archaea (*Nitrosopumilaceae* and *Nitrososphaeraceae*) occurred in several modules, implying a syntrophy relationship between Bathyarchaeota and nitrogen-cycling microorganisms. Ammonia-oxidizing archaea are keystone members that convert ammonia to nitrite and significantly influence nitrogen cycle in paddy soil (70). Bathyarchaeota harbor a series of genes related to the conversion of different nitrogen compounds to ammonium (60), and then ammonium can be further used as substrates for ammonia-oxidizing archaea. In addition, Genes involved in urea production also were observed in Bathyarchaeota genomes, and genes involved in urea degradation were found in the genomes of *Nitrososphaeraceae* (71). These findings indicate the interactions between Bathyarchaeota and nitrogen cycling microorganisms through urea and ammonium transformation. These co-occurrence patterns imply that Bathyarchaeota might be involved in nitrogen cycle in paddy soils. However, due to lack of pure culture, so far, the research on the metabolism of Bathyarchaeota are mainly based on genomic information. The specific metabolic pathways of Bathyarchaeota in paddy soil should be further confirmed by clear experimental evidences.

### Conclusion

Through a meta-analysis on the global *in situ* paddy soils sequencing data, we reveal a wide distribution and high abundance of Bathyarchaeota in paddy soils, and Bathy-6 is the predominant group. In addition, MAT and MAP are the main factors affecting the relative abundance of Bathyarchaeota and shaping bathyarchaeotal community structure. Recurrence of the associations between Bathyarchaeota and microorganisms involved in carbon and nitrogen metabolism suggested that Bathyarchaeota might be involved in diverse biogeochemical cycling in paddy soils. This study provides novel understanding of Bathyarchaeota in paddy soils at a global scale, and extends the research on Bathyarchaeota in arable soils other than sediments.

## SUPPLEMENTAL MATERIAL

Supplemental tables msystems.00143-23-s0001.xlsx Tables S1 to S3.

Supplemental figures msystems.00143-23-s0002.docx Fig. S1 to S5.

ASM does not own the copyrights to Supplemental Material that may be linked to, or accessed through, an article. The authors have granted ASM a non-exclusive, world-wide license to publish the Supplemental Material files. Please contact the corresponding author directly for reuse.
