# Supplementary material for: Global biogeographic distribution of Bathyarchaeota in paddy soils
Source: mSystems. 2023 May 29;8(3):e00143-23. doi: 10.1128/msystems.00143-23 (PMC10308895; doi:10.1128/msystems.00143-23)
Supplement: Supplemental figures — Fig. S1 to S5. [file msystems.00143-23-s0002.docx]

Global biogeographic distribution of Bathyarchaeota in paddy soil

Shu-Dan Xue^a,b^, Xing-Yun Yi^a,c^, Hui-Ling Cui^a,b^, Meng Li^d,e^, Jing-Jing Peng^f^, Yong-Guan Zhu^a,b,g^, Gui-Lan Duan^a,b#^

^a^State Key Lab of Urban and Regional Ecology, Research Center for Eco-Environmental Sciences, Chinese Academy of Sciences, Beijing 100085, China

^b^University of Chinese Academy of Sciences, Beijing 100049, China

^c^Department of plant and Environmental Sciences, University of Copenhagen, Thorvaldsensvej 40, 1871 Frederiksberg, Denmark

^d^Archaeal Biology Center, Institute for Advanced Study, Shenzhen University, Shenzhen, Guangdong, China

^e^Shenzhen Key Laboratory of Marine Microbiome Engineering, Institute for Advanced Study, Shenzhen University, Shenzhen, Guangdong, China

^f^College of Resources and Environmental Sciences, China Agricultural University, Beijing, China.

^g^Institute of Urban Environment, Chinese Academy of Sciences, Xiamen 361021, China

Running Title: Climate drives Bathyarchaeota community in paddy soils

#Address correspondence to Gui-Lan Duan, duangl@rcees.ac.cn


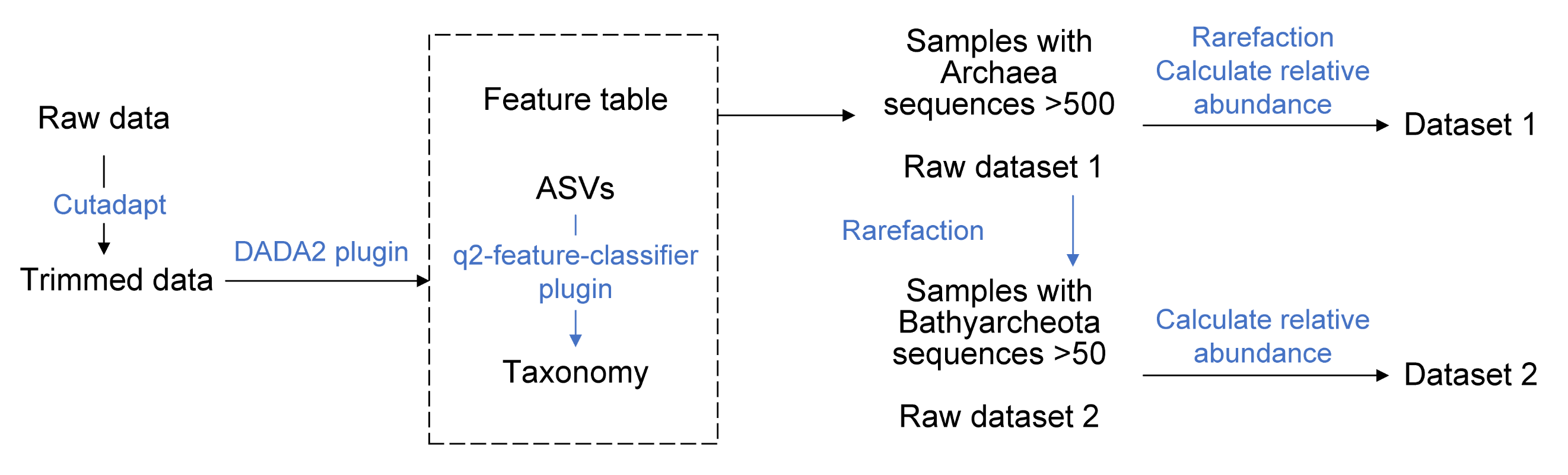


**Figure. S1.** The process of bioinformatics analysis and the corresponding obtained dataset at each step.


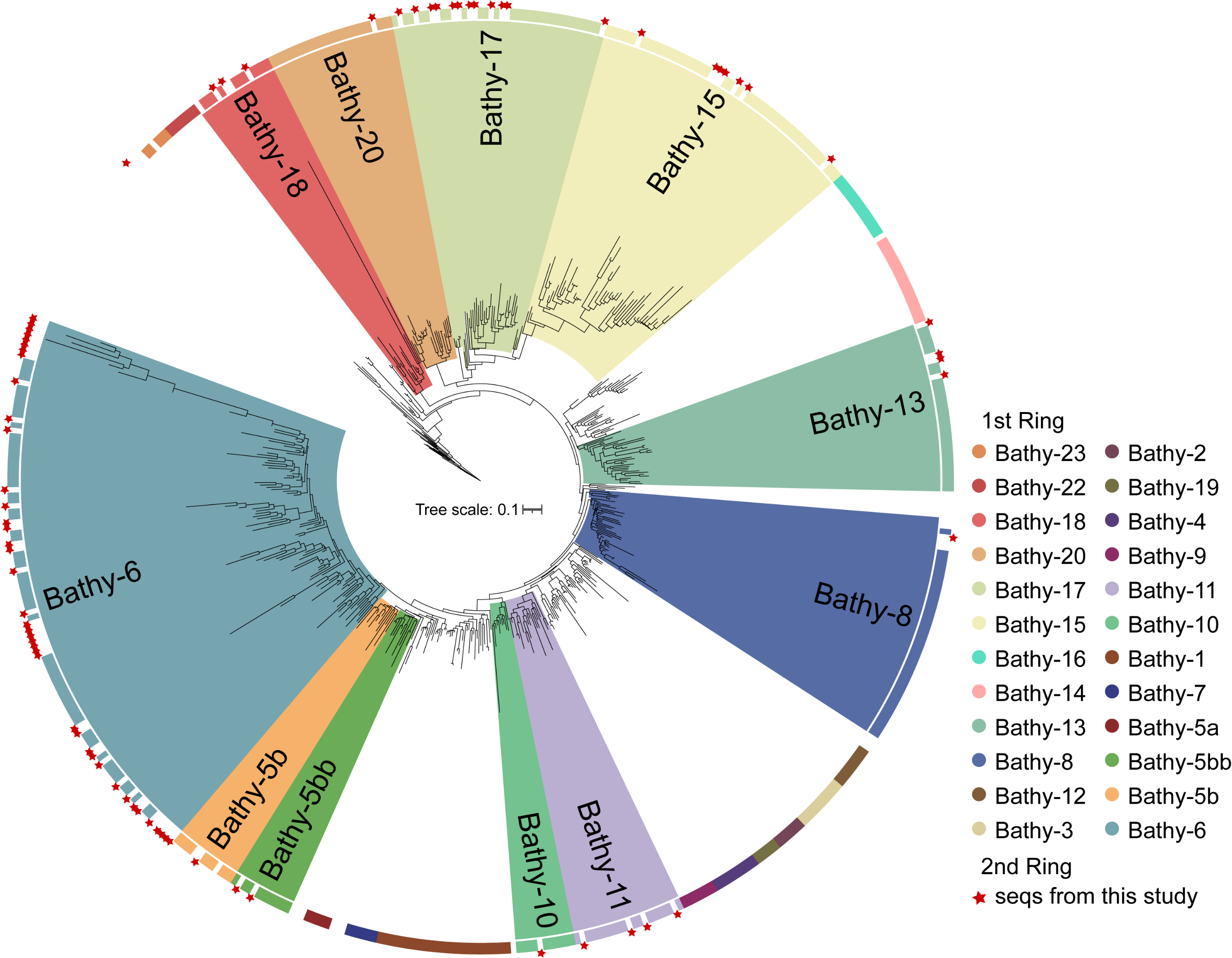


**Figure. S2.** Phylogenetic tree of Bathyarchaeota based on 16S rRNA amplicon datasets. The color stripes represented different bathyarchaeotal subgroups. The sequences marked with red asterisks were from this study.


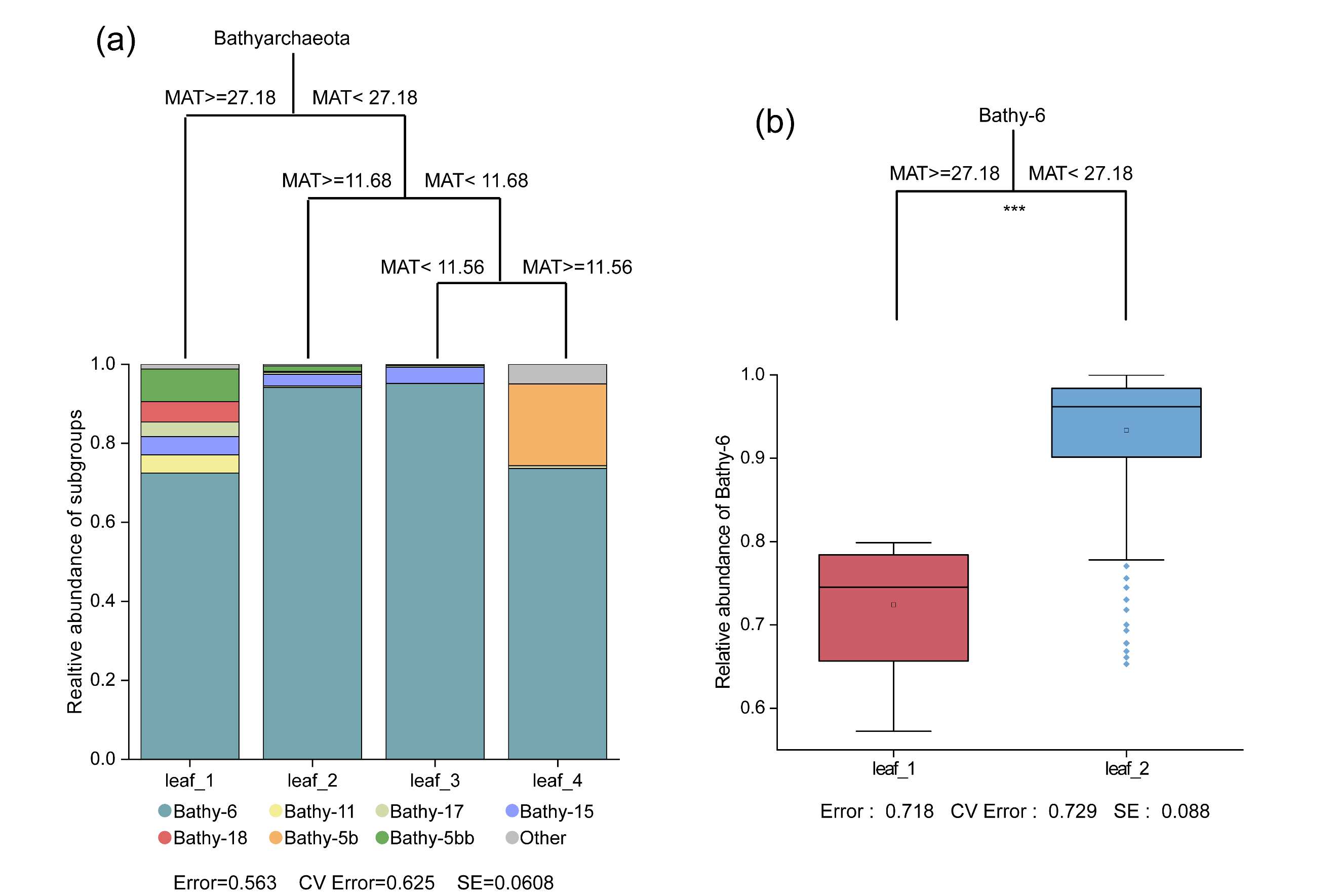


**Figure. S3.** Multivariate regression tree (MRT) analysis showed the relationships between the relative abundance of Bathyarchaeota and environmental factors collected from corresponding studies and WorldClim database, including pH, organic matter content, MAT, MAP and sample condition. (a) the whole bathyarchaeotal community; (b) subgroup: Bathy-6.


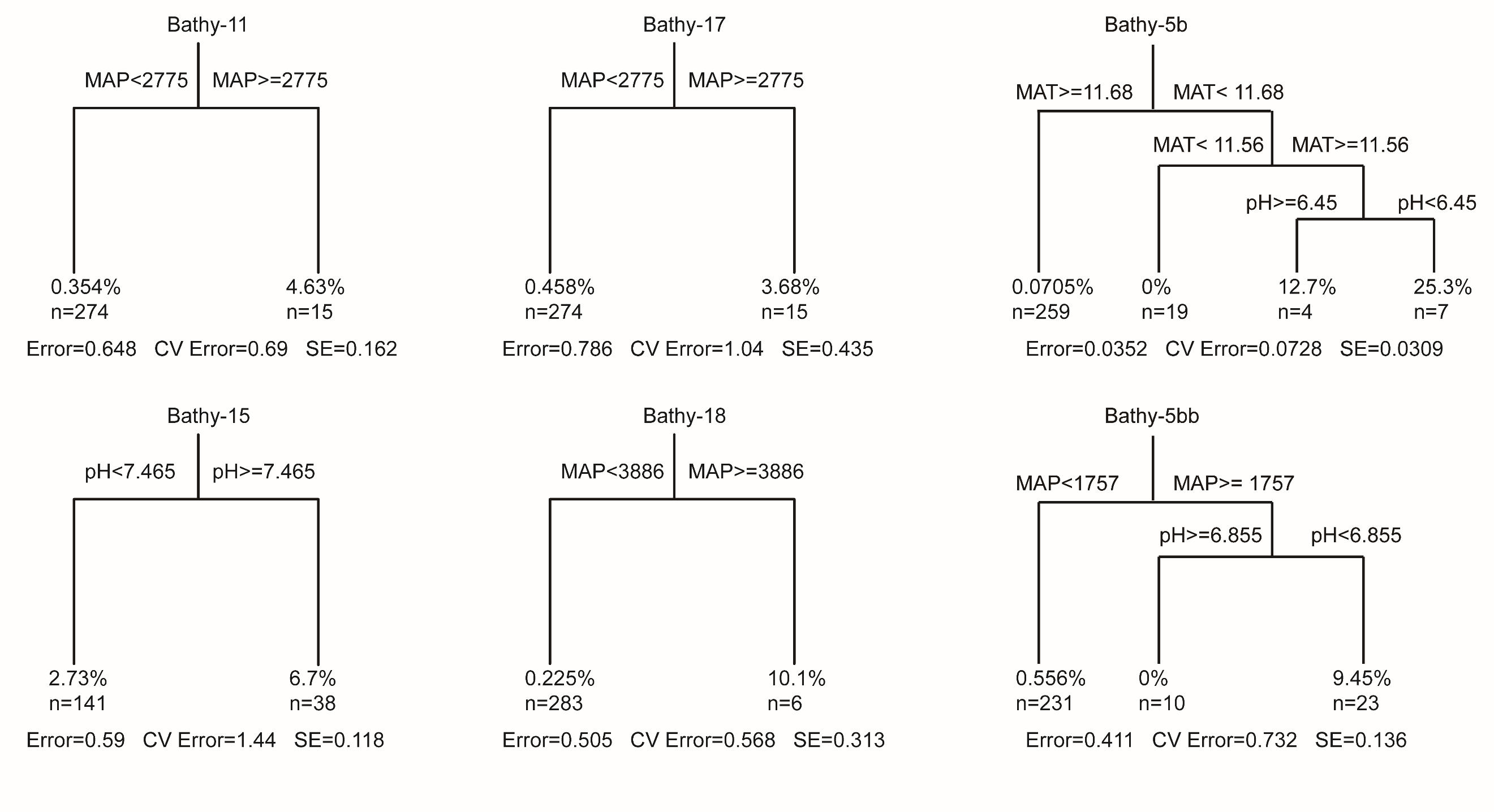


**Figure. S4.** Multivariate regression tree (MRT) analysis showed the relationships between the relative abundance of bathyarchaeotal subgroups and environmental factors.


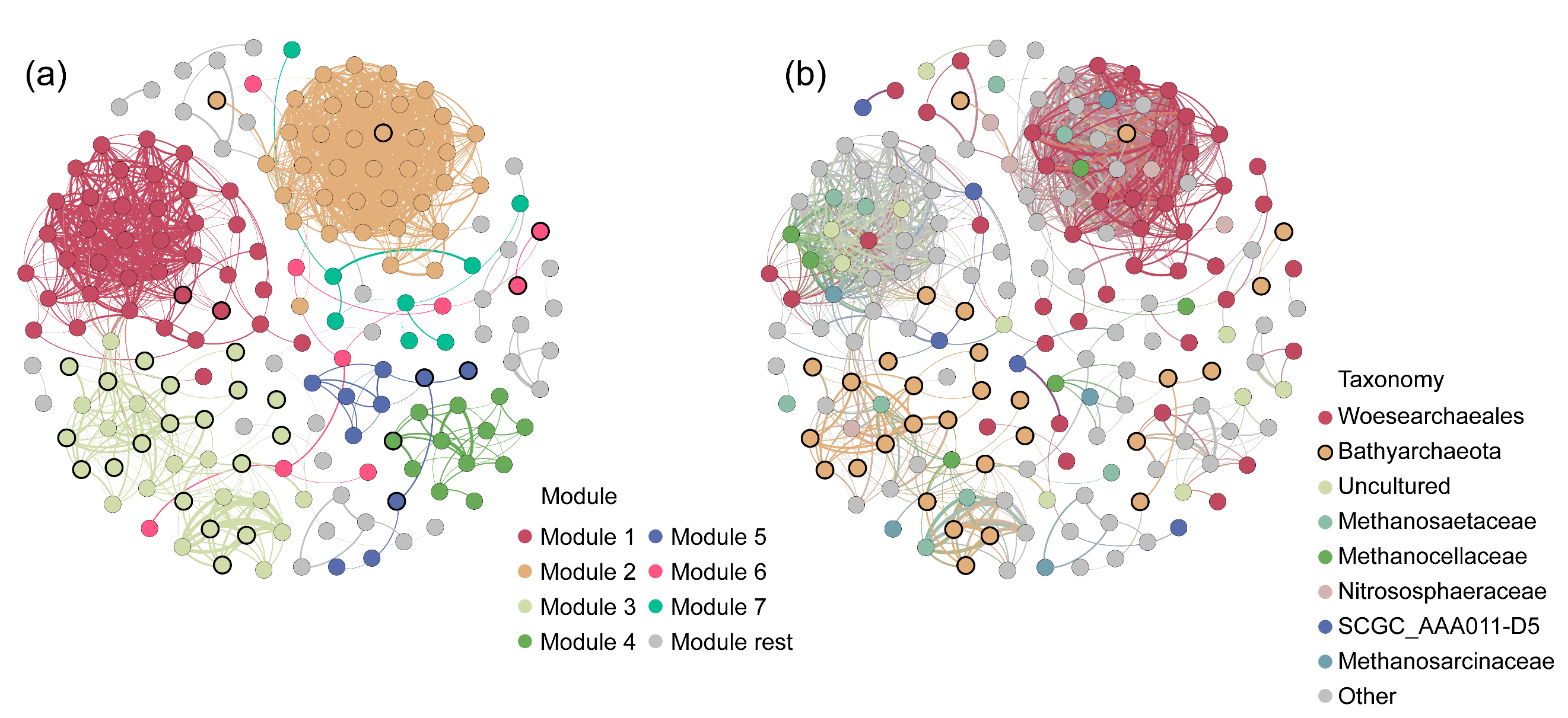


**Figure. S5.** Co-occurrence networks of archaeal lineages. (a) Nodes colored based on different modules. The nodes belonging to Bathyarchaeota were highlighted by using thicker border; (b) Nodes colored based on taxonomic classification.
